# Redox-dependent hydrogen-bond network rearrangement of ferredoxin–NADP^+^ reductase revealed by high-resolution X-ray and neutron crystallography

**DOI:** 10.1107/S2053230X25000524

**Published:** 2025-02-06

**Authors:** Midori Uenaka, Yusuke Ohnishi, Akane Ise, Jiang Yu, Naomine Yano, Katsuhiro Kusaka, Hideaki Tanaka, Genji Kurisu

**Affiliations:** a Protein Crystallography Laboratory, Institute for Protein Research Osaka University Suita Osaka565-0871 Japan; b Department of Biological Sciences, Graduate School of Science Osaka University Toyonaka Osaka560-0043 Japan; c Department of Macromolecular Science, Graduate School of Science Osaka University Toyonaka Osaka560-0043 Japan; d Structural Biology Division Japan Synchrotron Radiation Research Institute Hyogo679-5198 Japan; e Neutron Industrial Application Promotion Center Comprehensive Research Organization for Science and Society Tokai Ibaraki319-1106 Japan; Osaka University, Japan

**Keywords:** ferredoxin–NADP^+^ reductase, redox-dependent structural changes, hydrogen-bond networks, neutron crystallography, X-ray crystallography

## Abstract

High-resolution X-ray and neutron crystallography were employed to elucidate redox-dependent structural changes in ferredoxin–NADP^+^ reductase (FNR) from maize. This study focused on the rearrangement of hydrogen-bond networks upon FAD reduction. The X-ray structures of wild-type FNR in oxidized and reduced states were refined to 1.15 and 1.10 Å resolution, respectively, revealing no large structural changes in the main-chain backbones. Neutron crystallography provided complementary insights, confirming protonation at N1 and N5 of the isoalloxazine ring and visualizing hydrogen bonds that were undetectable by X-ray analysis. These findings illuminate the dynamic reorganization of water-mediated hydrogen-bond networks during redox transitions, which may underpin the redox-dependent modulation of partner binding by FNR. This integrated structural approach highlights the synergistic use of X-ray and neutron crystallography in studying redox-active proteins.

## Abstract 2

High-resolution X-ray and neutron crystallography reveal redox-dependent rearrangements in the hydrogen-bond network of ferredoxin–NADP^+^ reductase (FNR), highlighting structural changes around the FAD cofactor and shifts in water molecules that underpin its electron-transfer mechanism. These findings provide valuable insights into the functional modulation of FNR.

## 1. Introduction

The photosynthetic electron-transport chain on the thylakoid membrane consists of three integral membrane-protein complexes: photosystem II (PS II), cytochrome *b*_6_*f* and photosystem I (PS I). These complexes are electronically linked by small electron carriers, including plastoquinone, plastocyanin and ferredoxin (Fd). The electron-transfer process, initiated by light absorption, starts with oxidation of water by PS II and concludes with reduction of Fd by PS I. Reduced Fd then delivers electrons to various Fd-dependent enzymes, such as ferredoxin–NADP^+^ reductase (FNR), sulfite reductase (SiR), nitrite reductase (NiR), glutamate synthase and ferredoxin–thioredoxin reductase (Knaff, 1996 ▸; Hanke & Mulo, 2013 ▸; Goss & Hanke, 2014 ▸). Among these, FNR is the primary consumer of electrons from Fd, producing reducing equivalents in chloroplasts or cyanobacterial cells.

FNR is a flavoenzyme that contains a noncovalently bound flavin adenine dinucleotide (FAD) as a prosthetic group. It catalyzes redox reactions between Fd and NAD(P)H. The redox-active component is the isoalloxazine ring of FAD, which undergoes transitions among three states: fully oxidized (FAD), semiquinone (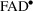; partially reduced) and hydroquinone (FADH_2_; fully reduced). Plant-type FNR is not only found in photosynthetic tissues such as leaves but also in nonphotosynthetic tissues such as roots and fruits, where tissue-specific isozymes are expressed: leaf-type FNR (L-FNR) in leaves and root-type FNR (R-FNR) in roots and fruits (Onda *et al.*, 2000 ▸; Hanke *et al.*, 2004 ▸). In root plastids, which lack PS I, R-FNR reduces nonphotosynthetic Fd by oxidizing NADPH from the oxidative pentose phosphate pathway (Flores *et al.*, 2005 ▸; Yonekura-Sakakibara *et al.*, 2000 ▸; Bowsher *et al.*, 1993 ▸). This reaction operates in reverse compared with the chloroplast FNR counterpart.

The interaction between Fd and Fd-dependent enzymes has been extensively studied using biochemical and biophysical methods. Studies have revealed that these interactions lack a consensus sequence or motif for Fd-dependency, and the critical residues on the surface of Fd vary among the partner enzymes (Hanke & Mulo, 2013 ▸; Hanke *et al.*, 2008 ▸; Kim *et al.*, 2016 ▸; Sakakibara *et al.*, 2012 ▸; Glauser *et al.*, 2004 ▸). Structural studies using X-ray crystallography, NMR spectroscopy and thermodynamic analysis show that Fd and its partner proteins form a transient but specific 1:1 complex primarily through electrostatic interactions (Kurisu *et al.*, 2001 ▸; Shinohara *et al.*, 2017 ▸; Kinoshita *et al.*, 2017 ▸; Chikuma *et al.*, 2021 ▸). These complexes must dissociate promptly after electron transfer to allow the next cycle, but the exact mechanism underlying dissociation upon electron transfer remains unclear. For example, in *Equisetum arvense* reduced Fd binds to oxidized FNR with a *K*_m_ of 0.62 µ*M*, while oxidized Fd binds to reduced FNR with a *K*_m_ of 11.4 µ*M*, indicating significant affinity changes depending on its redox state (Kurisu *et al.*, 2005 ▸; Teshima *et al.*, 2003 ▸).

High-resolution X-ray structures of L-FNR and R-FNR, complexed with nicotinamide, NADP^+^ or NADPH, have elucidated the mechanism of hydride transfer between NADPH and FAD (Deng *et al.*, 1999 ▸; Kean *et al.*, 2017 ▸). However, NADP(H) soaking was only applicable to crystals of FNR mutants that replaced the C-terminal tyrosine stacking with the isoalloxazine ring of FAD, and these studies could not determine the positions of the H atoms of wild-type FNR due to the limitations of X-ray crystallography. Since H atoms have only one electron, their scattering signals are significantly weaker than those of heavier atoms such as carbon, nitrogen and oxygen. Ultrahigh resolution (<1.2 Å) is required to visualize hydrogen positions, but achieving such a resolution often necessitates intense X-ray irradiation, which can damage crystals and inadvertently reduce redox proteins during data collection (Garman, 2010 ▸; Ohnishi *et al.*, 2020 ▸). In contrast, neutron crystallography offers a powerful alternative for locating H and D atoms in protein structures. Neutrons interact with atomic nuclei rather than electrons, making their scattering signals for hydrogen and deuterium comparable to those of heavier atoms. However, neutron crystallography requires large crystals (>1 mm^3^) and long exposure times due to the relatively weak intensity of neutron beams (Niimura & Podjarny, 2011 ▸).

Previous studies of the redox-dependent structural changes of FNR have focused on the C, N and O atoms around the isoalloxazine ring of FAD(H_2_). However, the addition of two hydrogens to N1 and N5 of the ring during reduction has not been thoroughly investigated. To address this gap, we employed high-resolution X-ray and neutron crystallography to analyze structural changes, including the positions of H/D atoms, upon FAD reduction. This approach allowed us to confirm the protonation states of oxidized wild-type FNR (wtFNR_oxi_) and reduced wild-type FNR (wtFNR_red_) and the precise hydrogen-bond networks involving the surrounding amino acids and water molecules. This research was conducted using R-FNR from maize.

## 2. Materials and methods

### 2.1. Preparation, crystallization, X-ray diffraction experiments and structure refinement

#### 2.1.1. Preparation of wild-type R-FNR from *Zea mays*

The wild-type R-FNR protein was expressed in *Escherichia coli* and purified using the method described previously (Onda *et al.*, 2000 ▸). The protein was concentrated to 60 mg ml^−1^ using an Amicon Ultra 30K (Millipore, USA), flash-frozen in liquid nitrogen and stored at −80°C for future use.

#### 2.1.2. Crystallization and cryoprotection of wtFNR_oxi_ for X-ray crystallography

An R-FNR crystal was initially obtained using the hanging-drop vapor-diffusion method. The protein stock solution was diluted to a concentration of 30 mg ml^−1^ in a buffer consisting of 50 m*M* Tris–HCl pH 7.5, 150 m*M* NaCl. It was then mixed with an equal volume of precipitant solution [0.2 *M* 2-(*N*-morpholino)ethanesulfonic acid (MES) pH 6.0, 24.6% PEG 2000] and equilibrated against the precipitant solution at 20°C. All crystals used for X-ray diffraction experiments were prepared using the batch method with microseeds. The microseed solution was prepared by breaking a macro-scale R-FNR crystal (∼300 µm) in stabilization buffer (0.24 *M* MES pH 6.4, 29.5% PEG 2000) using a Seed Bead Kit (Hampton Research). The crystallization drops for the batch method with microseeds were prepared by mixing 1 µl R-FNR solution (30 mg ml^−1^ in 50 m*M* Tris–HCl pH 7.5, 150 m*M* NaCl buffer), 0.8 µl precipitant solution (0.195 *M* MES pH 6.0, 23.985% PEG 2000) and 0.2 µl of the microseed solution diluted in stabilization buffer. The drop was then completely covered with paraffin oil and incubated at 20°C. After 2–3 days, crystals of approximately 300 × 300 × 100 µm were obtained. The crystals were soaked in a cryoprotectant consisting of 0.24 *M* MES pH 6.4, 15% PEG 2000 and glycerol. The glycerol concentration was initially 1% and was increased by 1% every 5 min until it reached 20%. The crystals were then mounted using CryoLoops (Hampton Research) and cryocooled in liquid nitrogen.

#### 2.1.3. Dithionite soaking of a wtFNR_red_ crystal

All steps described in this section were performed in an anaerobic chamber. All liquids and the crystallization drop containing R-FNR crystals were stored under anaerobic conditions for one day prior to starting the experiment. An oxidized R-FNR crystal (∼300 × 300 × 100 µm) was soaked in 50 µl of a dithionite-containing buffer (5 m*M* sodium dithionite, 0.2 *M* MES pH 6.0, 15% glycerol) for 5–6 h. The reduced crystal was then mounted using a CryoLoop (Hampton Research) and cryocooled in liquid nitrogen.

#### 2.1.4. Micro-spectrophotometry of a wtFNR crystal

The absorption spectrum of the R-FNR crystal mounted on a CryoLoop was measured using an offline micro-spectrophotometer at SPring-8/RIKEN equipped with a DT-Mini light source and an SD2000 detector (Ocean Optics Inc.). To detect spectral changes induced by X-ray exposure, an X-ray beam with a wavelength of 1.0000 Å from BL26B2 at SPring-8 (Ueno *et al.*, 2005 ▸, 2006 ▸) was used. The beam was shaped by a 100 µm pinhole. For this beam size, the flux was 3.84 × 10^10^ photons s^−1^. The full-width at half-maximum (FWHM) of the beam was 86 µm horizontally and 93 µm vertically. Micro-spectrophotometry was conducted following irradiation with a fixed dose of X-rays (1.3 MGy). Subsequently, the same dose of X-rays was applied and the absorption spectrum was measured again. This procedure was repeated iteratively until no further changes in the absorption spectrum were observed. The X-ray dose absorbed by the crystals was calculated using *RADDOSE*-3*D* (Zeldin *et al.*, 2013 ▸). Since the changes in the absorption spectrum of a mounted crystal depend on the absorbed X-ray dose, the size of the beam from the optical fiber was used for dose calculations in *RADDOSE*-3*D*. As *RADDOSE*-3*D* does not support circular beam shapes, the beam was approximated as a square with an area equal to the cross-sectional area of the light for dose estimation.

#### 2.1.5. X-ray diffraction experiments and analysis of wtFNR_oxi_ and wtFNR_red_ crystals

X-ray diffraction experiments were conducted on BL44XU at SPring-8 using an EIGER X 16M detector to detect diffraction spots. A focused X-ray beam (λ = 0.9000 Å) was used for these diffraction experiments. The beam size was adjusted using a 50 µm pinhole and the beam flux was attenuated with 1.6 mm aluminium foil. To prevent X-ray radiation damage, data were collected in six non-overlapping angular shells from a single crystal. X-ray data collection started at one end of the crystal. A total of 200 images covering 20° of rotation were collected from a single assigned point. The measurement point was then moved 60 µm towards the opposite end and another 20 images were collected from a fresh part of the crystal. This process of crystal movement and data collection was repeated until the entire angular range was covered using six assigned exposure points. All images were processed with *XDS* (Kabsch, 2010 ▸) and the six data sets were scaled and merged using *XSCALE*. Data-collection statistics were calculated with *AIMLESS* (McCoy *et al.*, 2007 ▸). Initial phase information was obtained using molecular replacement in *Phaser* (Afonine *et al.*, 2012 ▸), using the crystal structure of R-FNR (PDB entry 5h59; Shinohara *et al.*, 2017 ▸) as the search model. Iterative restrained refinement of *XYZ* coordinates, real-space refinement, isotropic *B*-factor refinement, TLS refinement and manual model correction were performed starting from the initial model using *phenix.refine* (Adams *et al.*, 2010 ▸) and *Coot* (Emsley *et al.*, 2010 ▸). The *F*_o_(wtFNR_oxi_) − *F*_o_(wtFNR_red_) difference maps were generated with *FFT* in the *CCP*4 suite (Agirre *et al.*, 2023 ▸) using phase angles calculated from the final refined structure against the oxidized data set to confirm structural differences between the two data sets.

### 2.2. Preparation of crystals for neutron crystallography

#### 2.2.1. Preparation of a large crystal of wtFNR_oxi_ for neutron crystallography

A large crystal of wtFNR for neutron crystallography was prepared using the batch method with microseeds. Reagents containing heavy water (99.9% D_2_O) were used during crystallization to facilitate H/D exchange in the protein and to reduce incoherent background scattering from H atoms in the diffraction pattern. The purified protein solution was exchanged into a buffer consisting of 50 m*M* Tris–HCl pD 7.91, 150 m*M* NaCl in D_2_O and diluted to 30 mg ml^−1^. The microseed solution was prepared using the same method as used for X-ray diffraction experiments, but with reagents containing heavy water (99.9% D_2_O). Silicone-coated Durham tubes (6 × 30 mm, Maruemu Corporation) were filled with 300 µl Fluorinert. Fluorinert, an inert solvent with a specific gravity of 1.7, is heavier than the crystallization solution and does not mix with it. The crystallization solution was layered on top of the Fluorinert to prevent the crystals from sticking to the walls of the Durham tube during growth. The crystallization solution for the batch method with microseeds was prepared by mixing 100 µl R-FNR solution (30 mg ml^−1^ in 50 m*M* Tris–HCl pD 7.91, 150 m*M* NaCl buffer in D_2_O), 80 µl precipitant solution (0.195 *M* MES pH 6.0, 23.985% PEG 2000 in D_2_O), 18.8 µl stabilization buffer and 0.2 µl microseed solution diluted in stabilization buffer. Additionally, 200 µl paraffin oil was layered on top of the crystallization solution to prevent drying. The crystals were allowed to grow at 20°C for 3–4 weeks. The crystals were soaked in a cryoprotectant consisting of 0.24 *M* MES pH 6.4, 15% PEG 2000 and glycerol. The glycerol concentration was initially set to 1% and was increased by 1% every 10 min until it reached 20%. In the final stage, the crystals were soaked in the solution for an extended period, including 5–6 h in a dithionite-containing buffer. The crystals were then mounted on a thin nylon loop (3.0 mm in diameter) and cooled in liquid nitrogen.

#### 2.2.2. Preparation of a large crystal of wtFNR_red_ for neutron crystallography

All steps described in this section were performed in an anaerobic chamber. All liquids and the crystallization drop containing R-FNR crystals were stored under anaerobic conditions for one day prior to starting the experiment. A large wtFNR_oxi_ crystal (∼3 × 2 × 1 mm) was soaked in 100 µl of a dithionite-containing buffer (5 m*M* sodium dithionite, 0.2 *M* MES pD 6.41 and glycerol). The glycerol concentration was initially set to 0% and was increased by 1% every 10 min until it reached 20%. At the 20% glycerol step, the crystals were soaked in the solution for an extended period, with a total soaking time of 5–6 h in the dithionite-containing buffer. The crystals were then mounted on a thin nylon loop (3.0 mm in diameter) and cooled in liquid nitrogen. The absorption spectrum from the edge of a large wtFNR_red_ crystal mounted on the CryoLoop was measured using an offline micro-spectrophotometer at SPring-8/RIKEN equipped with a DT-Mini light source and an SD2000 detector (Ocean Optics Inc.). As the large crystals were too thick to measure at the center, measurements were taken at the edge of the crystal.

### 2.3. Neutron diffraction experiments and structure refinement

#### 2.3.1. Neutron diffraction experiments

Time-of-flight neutron diffraction data for oxidized and reduced R-FNR crystals were collected using the BL03 IBARAKI Biological Crystal Diffractometer (iBIX) at the Materials and Life Science Experimental Facility (MLF) of the Japan Proton Accelerator Research Complex (J-PARC; Tanaka *et al.*, 2009 ▸, 2010 ▸; Kusaka *et al.*, 2013 ▸). The instrument was equipped with 34 two-dimensional position-sensitive detectors utilizing a scintillator sheet and wavelength-shifting fiber. Data collection was conducted at 100 K (Hosoya *et al.*, 2009 ▸). Both oxidized and reduced crystals were measured under the same conditions. The accelerator power of the proton beam for the spallation neutron source was 730 kW. A total of 24 data sets were collected using a circular beam with a diameter of 3 mm and a selected neutron wavelength range of 2.05–5.38 Å. Data reduction was performed using *STARGazer* 3.8.2 (Yano *et al.*, 2018 ▸; Ohhara *et al.*, 2009 ▸), which employs the elliptical col method for peak integration (Kusaka *et al.*, 2020 ▸). We determined a resolution limit of 1.8 Å based on visual inspection of the resultant density maps together with the *I*/σ(*I*) values in the highest resolution shell, completeness and multiplicity values.

#### 2.3.2. Structure refinement

Model refinement was performed using *REFMAC* (Yamashita *et al.*, 2023 ▸; Catapano *et al.*, 2023 ▸) and *Coot* (Emsley *et al.*, 2010 ▸), utilizing only the neutron diffraction data and referencing the high-resolution X-ray structures obtained in this study (oxidized form, PDB entry 9kkg; reduced form, PDB entry 9kkh). The neutron diffraction data set was converted to an MTZ file using *ImportScaled* in the *CCP*4 suite (Agirre *et al.*, 2023 ▸). 5% of the reflections were flagged as the free-*R* set. After several cycles of refinement, during which the model was modified in *Coot*, neutron scattering length density (NSLD) peaks potentially corresponding to H and D atoms were observed in the *mF*_o_ − *DF*_c_ NSLD difference map. H and D atoms were placed in the model using *phenix.readyset*. Exchangeable hydrogen sites were treated as disordered models with H and D atoms, and their initial occupancies were set to 0.5/0.5. Subsequently, exchangeable H atoms other than those in the main chains were removed from the atomic coordinates. H and D atoms at exchangeable sites in protein side chains, solvent water molecules and ligands were manually added to the atomic coordinates and refined. H and D atoms were included in the model when corresponding peaks were observed in the *mF*_o_ − *DF*_c_ NSLD difference map. This process of modeling and refinement was repeated until all observable H and D atoms had been included in the model. All polar hydrogens were treated as H1/D exchangeable hydrogens, and their deuterium fractions were refined individually. The positions of all H and D atoms were refined with restraints based on the monomer library in *CCP*4 (Agirre *et al.*, 2023 ▸). All molecular figures were generated using *PyMOL* (Schrödinger).

## 3. Results

### 3.1. Preparing reduced FNR crystals via dithionite soaking

Since recombinant FNR primarily exists in its oxidized state after purification due to its redox potential, it was crucial to verify spectroscopically whether chemically reduced crystals were fully reduced. We employed dithionite soaking to reduce the crystals. Small, oxidized crystals (∼300 × 300 × 100 µm) prepared for X-ray diffraction experiments were soaked in a 5 m*M* dithionite-containing buffer under anaerobic conditions for 5–6 h, following optimization of the soaking time. The absorption spectrum of fully reduced FADH_2_ exhibits a characteristic peak at approximately 420 nm. Spectroscopic measurements using a micro-spectrophotometer revealed a single peak at around 420 nm in these crystals, confirming that the wtFNR was fully reduced to the two-electron reduced state wtFNR_red_ (Supplementary Fig. S1*a*). For neutron diffraction experiments, larger oxidized crystals (∼3 × 2 × 1 mm) were soaked under the same conditions. Absorption spectra from these crystals also displayed a single peak near 420 nm, indicating successful reduction (Supplementary Fig. S2).

### 3.2. Estimating the X-ray dose to minimize the unavoidable reduction of oxidized FNR crystals

Previous X-ray structural studies of oxidized FNR have not accounted for the potential partial reduction caused by X-ray irradiation. To address this, we conducted micro-spectroscopic measurements to assess the reduction rate under incremental X-ray irradiation. The spectra of FNR crystals changed progressively with increasing X-ray dose but saturated after 3.9 MGy of irradiation (Supplementary Fig. S1*b*). The strong absorbance at 460 nm, characteristic of the flavoenzyme, served as an indicator of reduction. The absorbance at 460 nm (*A*_460 nm_) before irradiation was defined as 0% reduction (FAD; fully oxidized) and that after 3.9 MGy irradiation as 100% reduction (FADH_2_). The reduction rate was proportional to the X-ray dose up to 1.3 MGy (Supplementary Fig. S1*c*). To limit the reduction rate to less than 10%, it was necessary to keep the X-ray dose below 0.11 MGy. Consequently, X-ray diffraction data for wtFNR_oxi_ crystals were collected using multiple irradiation points within a single crystal to minimize the dose. The absorbed dose during the diffraction experiment was calculated using *RADDOSE*-3*D* (Zeldin *et al.*, 2013 ▸) and estimated to be 0.097 MGy, meeting the required threshold.

### 3.3. High-resolution X-ray structures of oxidized and reduced wild-type and R115A FNR

**Figure 1:**
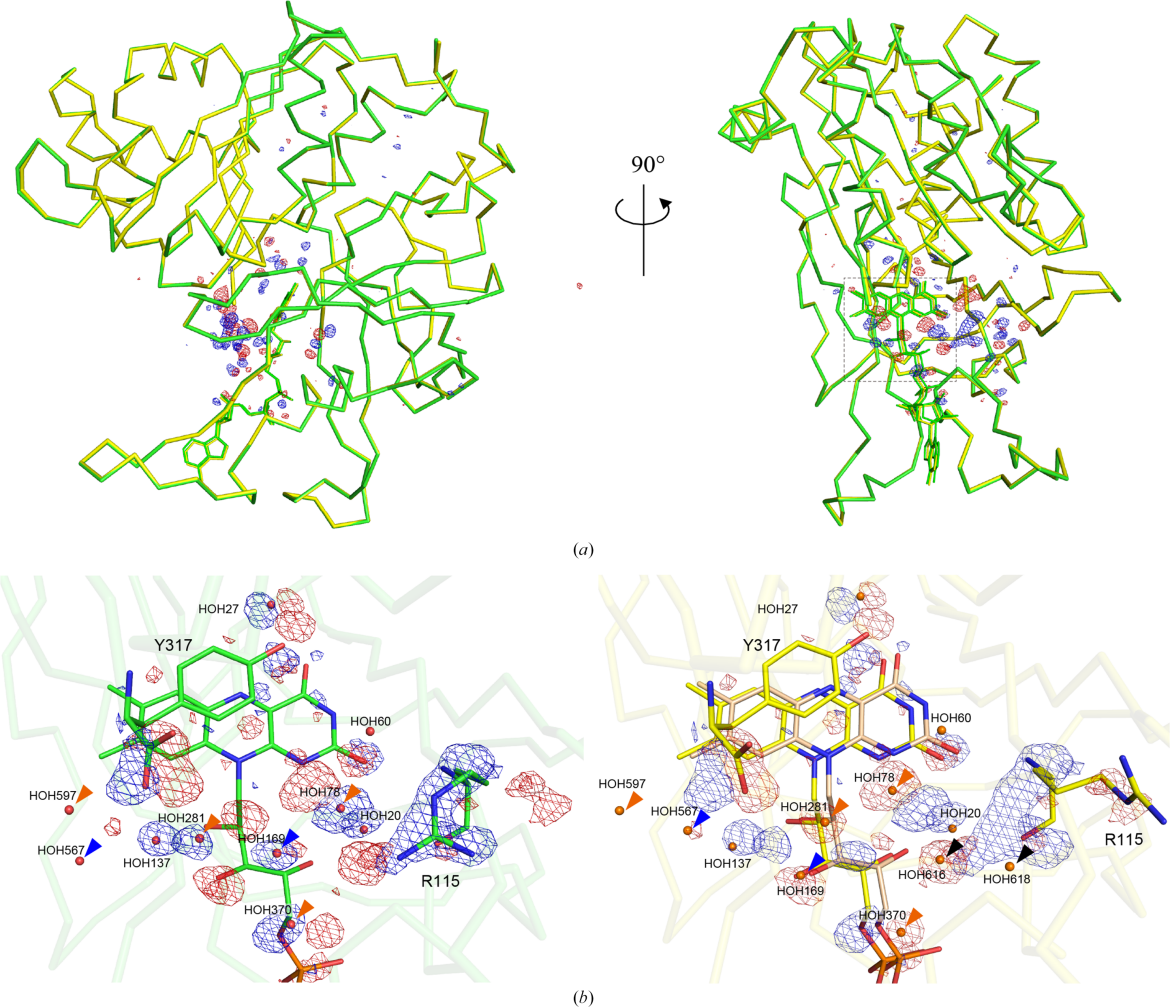
X-ray structures of wtFNR_oxi_ and wtFNR_red_. (*a*) Comparison of the overall structures of oxidized (green) and reduced (yellow) R-FNR. Each model was aligned based on the positions of all C^α^ atoms (backbone r.m.s.d. = 0.121 Å). The green and red meshes represent the *F*_o_(wtFNR_oxi_) − *F*_o_(wtFNR_red_) difference map contoured at +4.0σ and −4.0σ, respectively. (*b*) Enlarged views around FAD/FADH_2_. The left panel shows the oxidized model with the difference map, while the right panel displays the reduced model with the same difference map. In the right panel, conformer *A* of FADH_2_ is shown in yellow and conformer *B* in pale orange. Orange arrows indicate moving water molecules. Among these, blue arrows highlight water molecules that shifted by more than 2 Å and black arrows represent newly appearing water molecules.

Crystals of both oxidized wild-type FNR (wtFNR_oxi_) and reduced wild-type FNR (wtFNR_red_) were determined to belong to the same space group, *P*3_2_21, with no significant differences in their unit-cell parameters (Table 1 ▸). The X-ray structures of wtFNRoxi and wtFNRred were refined to resolutions of 1.15 and 1.10 Å, respectively. The resolution achieved for the wtFNR_oxi_ structure in this study (1.15 Å) is higher than those of previously reported oxidized FNR structures available in the Protein Data Bank (PDB), which range from 1.351 to 1.953 Å (for example PDB entries 5h59, 5vw3, 5vw4, 5vw5, 5vw6, 5vw8, 5vw9, 5vwa and 5vwb). A comparison of the overall structures of wtFNR_oxi_ and wtFNR_red_ is shown in Fig. 1 ▸(*a*). The electron-density map of wtFNR_red_ indicated that FADH_2_ was not in a single conformation (Supplementary Fig. S3). Therefore, FADH_2_ was modeled as dual conformers. The occupancy of conformer *A*, the major conformer of FADH_2_, was refined to 60% occupancy. The isoalloxazine ring of conformer *B*, the minor conformer of FADH_2_, was displaced by 0.6 Å relative to that of conformer *A*. When compared with wtFNR_oxi_, the FAD conformation in wtFNR_oxi_ closely resembles that of conformer *A* in wtFNR_red_. The structural model of wtFNR_red_ with dual conformers is shown in Fig. 1 ▸(*b*) (right). The structure of R115A mutant FNR (mtFNR) was also determined in the oxidized form (mtFNR_oxi_) to examine the arrangement of water molecules around the FAD moiety. Although the space group of the mtFNR_oxi_ crystals differed (*P*3_1_21, Supplementary Table S1), the main-chain structure of mtFNR_oxi_ was highly similar to those of wtFNR_oxi_ and wtFNR_red_. Focusing on the water molecules surrounding FAD, the arrangement in the mtFNR_oxi_ structure closely resembled that in wtFNR_red_ (Supplementary Fig. S4). The crystallo­graphic statistics for the final models of wtFNR_oxi_, wtFNR_red_ and mtFNR_oxi_ were of high quality and were within acceptable limits (Table 1 ▸ and Supplementary Table S1).

### 3.4. Neutron diffraction experiment

**Figure 2:**
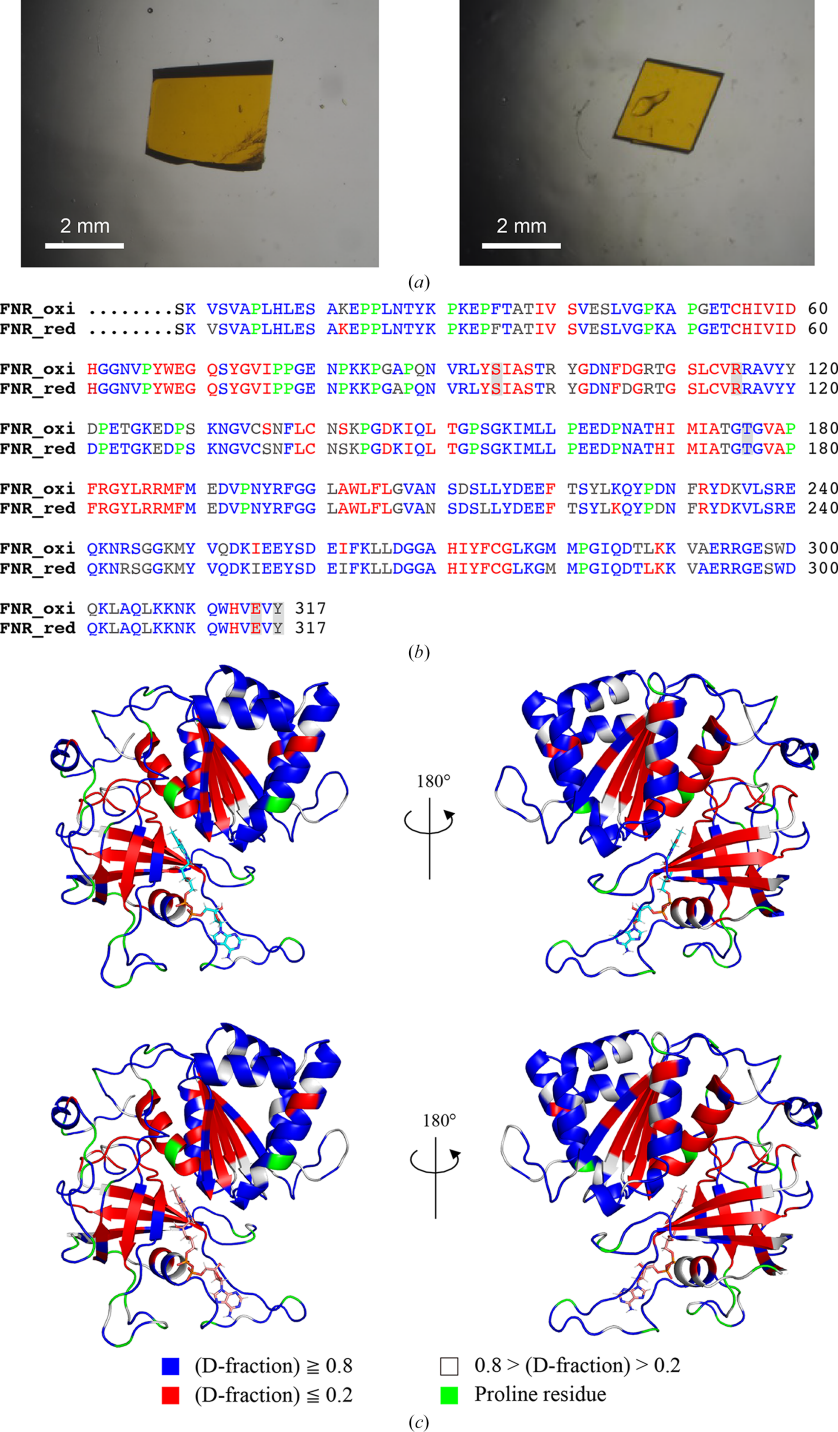
Neutron crystallography of wtFNR. (*a*) Large crystals used for neutron crystallography. The oxidized crystal is shown on the left and the reduced crystal is shown on the right. (*b*) Comparison of the H/D-substitution rates of amide protons for each residue in the sequence. Residues with a high H/D-substitution rate (over 80%) are colored blue, those with a low rate (under 20%) are colored red and residues with intermediate rates are colored gray. Proline residues are shown in green. (*c*) Models color-coded based on the H/D-substitution rate in the same color codes as in (*b*). The oxidized model is shown at the top and the reduced model is shown at the bottom.

After purification, H/D exchange was conducted by replacing the standard buffer with deuterated buffer. Crystals were then grown using solutions prepared with deuterated water and deuterated chemicals. For wtFNR_oxi_, a large crystal with a volume of 5.76 mm^3^ was grown aerobically and used for neutron data collection. To prepare wtFNR_red_ crystals, another large wtFNR_oxi_ crystal (volume of 1.98 mm^3^) was soaked in a dithionite solution under anaerobic conditions (Fig. 2 ▸*a*). Both crystals belonged to space group *P*3_2_21, with no significant differences in lattice constants. The resolution limit for neutron diffraction data was 1.80 Å for both oxidized and reduced crystals (Table 2 ▸). Refinement was performed exclusively using neutron structure factors due to the relatively high resolution of the neutron data. To ensure the accuracy of the structure, we used the high-resolution X-ray coordinates obtained in this study as a reference model and refined the structure by applying restraints on interatomic distances. The final neutron structure of wtFNR_oxi_ included 3234 H atoms, 1440 D atoms and 447 deuterated water molecules. Similarly, the neutron structure of wtFNR_red_ contained 3096 H atoms, 1367 D atoms and 413 deuterated water molecules. Additionally, the H/D-exchange ratios of main-chain amide H atoms were analyzed for both neutron structures. Comparisons of the H/D-exchange ratios between wtFNR_oxi_ and wtFNR_red_ revealed no significant differences (Figs. 2 ▸*b* and 2 ▸*c*).

### 3.5. Comparison of X-ray and neutron structures

**Figure 3:**
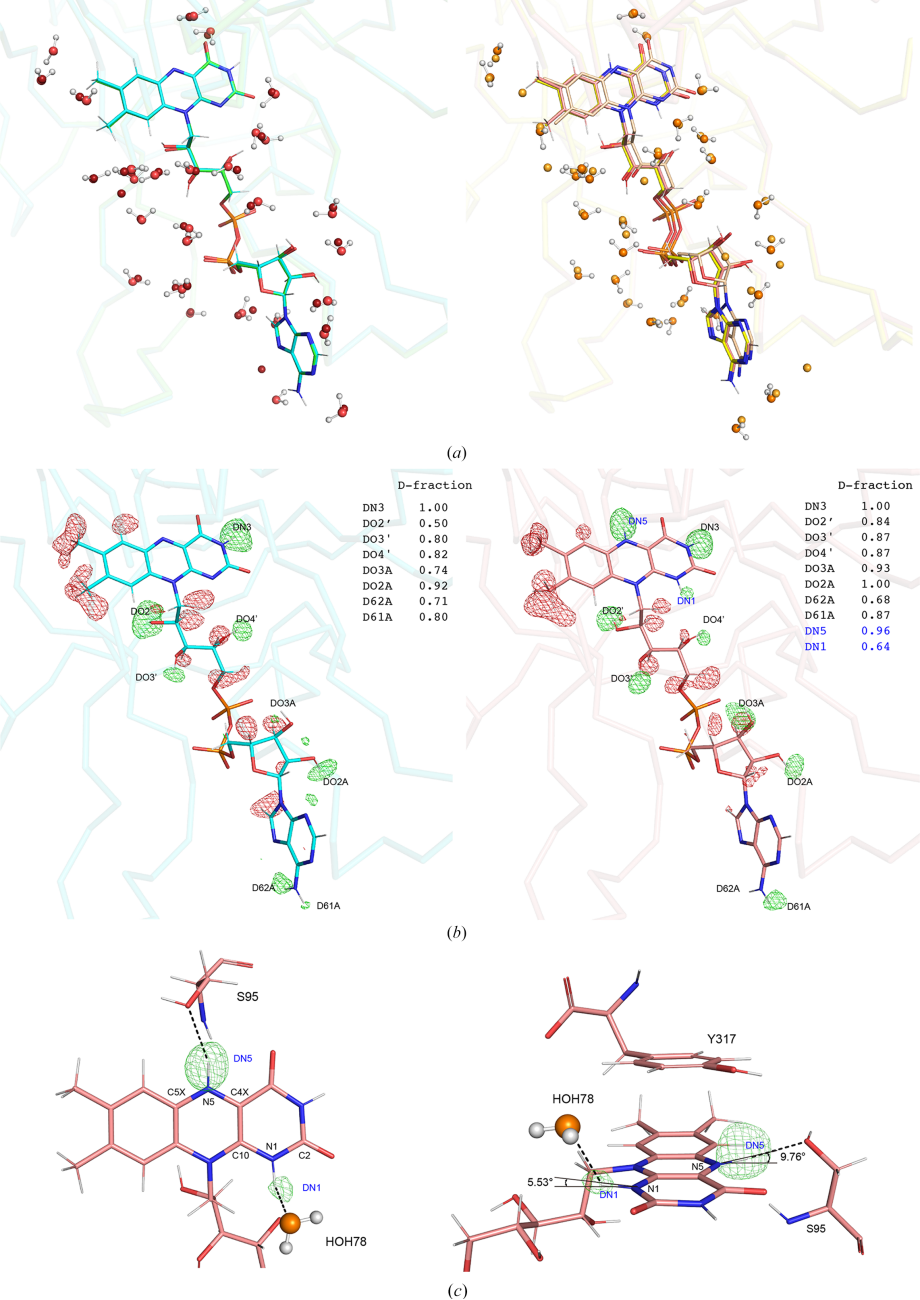
Neutron structures of oxidized and reduced R-FNR. (*a*) Comparison of X-ray and neutron structures around FAD/FADH_2_. Left: oxidized form, X-ray structure (model, green; water, dark red) and neutron structure (model, cyan; O atoms of water, red). Each model was aligned based on the positions of all C^α^ atoms (backbone r.m.s.d. = 0.102 Å). Right: reduced form, X-ray structure (conformer *A*, yellow; conformer *B*, pale orange; water, light orange) and neutron structure (model, salmon pink; O atoms of water, orange). Each model was aligned based on the positions of all C^α^ atoms (backbone r.m.s.d. = 0.158 Å). (*b*) Neutron maps around FAD/FADH_2_. The neutron *F*_o_ − *F*_c_ neutron omit scattering length density (SLD) map is contoured with positive (green) and negative (red) density at +3.2σ and −3.2σ, respectively. The oxidized form is shown on the left and the reduced form is shown on the right. D-fractions are also listed in the right corner. (*c*) Enlarged view of the isoalloxazine ring in the reduced-state neutron structure. The neutron *F*_o_ − *F*_c_ neutron omit SLD map of D atoms added during reduction is contoured with positive density (green) at +3.2σ. Black dashed lines indicate hydrogen bonds. The right panel shows the structure rotated by 90° along the *x* axis and 90° along the *y* axis.

The X-ray and neutron structures of both oxidized and reduced FNR were superimposed, ensuring that the positions of heavy atoms (C, N, O, S and P) aligned well. This alignment was expected, as the X-ray structures served as the reference models for neutron refinement (Fig. 3 ▸). Focusing on FADH_2_ in wtFNR_red_, the position of FADH_2_ in the neutron structure closely matched that of conformation *A* in the X-ray structure. This observation suggests that FADH_2_ predominantly exists in conformation *A*, while conformation *B* observed in the X-ray structure may represent an alternate state during the catalytic cycle. During refinement, water molecules in the neutron structure were not restrained to match those in the X-ray structures. This allowed an independent assessment of water-molecule positions, particularly those within 5 Å of the FAD/FADH_2_ moiety (Fig. 3 ▸*a*). The X-ray structures revealed six additional water molecules compared with the neutron structures, which is likely to be due to the higher resolution limit of the X-ray data. Importantly, all of the water molecules identified by neutron crystallography were also present in the X-ray models, confirming their structural relevance.

### 3.6. Protonation state of the isoalloxazine ring of FAD determined from the neutron structure

The protonation state of the isoalloxazine ring in wtFNR_oxi_ and wtFNR_red_ was analyzed using neutron crystallography. Differences between the oxidized and reduced structures were clearly visualized through *F*_o_ − *F*_c_ neutron omit maps, where H atoms of FAD/FADH_2_ were excluded during map calculation (Fig. 3 ▸*b*). In the wtFNR_oxi_ structure, no positive peaks corresponding to deuterium were observed around the N1 and N5 atoms of the isoalloxazine ring (Fig. 3 ▸*b*, left), indicating that FAD exists in the fully oxidized state. In contrast, the wtFNR_red_ structure displayed two additional positive peaks derived from deuterium near the N1 and N5 atoms, signifying that the FAD was reduced to FADH_2_ (Fig. 3 ▸*b*, right). The deuterium (D) fractions at these sites, DN1 and DN5, were calculated to be 0.64 and 0.96, respectively. The lower D fraction at DN1 suggests that the second reduction step, involving protonation of the N1 atom, may be incomplete in the crystalline state. The distinct D fractions at DN1 and DN5 highlight potential differences in the reduction environment or dynamics within the crystal. Another intriguing finding was the bonding angles of DN1 and DN5 relative to the planar isoalloxazine ring. The angle from the plane was 5.53° for DN1 and 9.76° (Supplementary Fig. S5) for DN5 (Fig. 3 ▸*c*). These oblique N—D bond formations appear to be constrained by N—D⋯O hydrogen bonds involving the O^γ^ atom of Ser95 or the O atom of HOH78, which are located above the iso­alloxazine ring. Meanwhile, the planar structure of FADH_2_ is maintained through π–π interactions with Tyr317.

### 3.7. Rearrangement of hydrogen bonds through the water molecules around FAD

**Figure 4:**
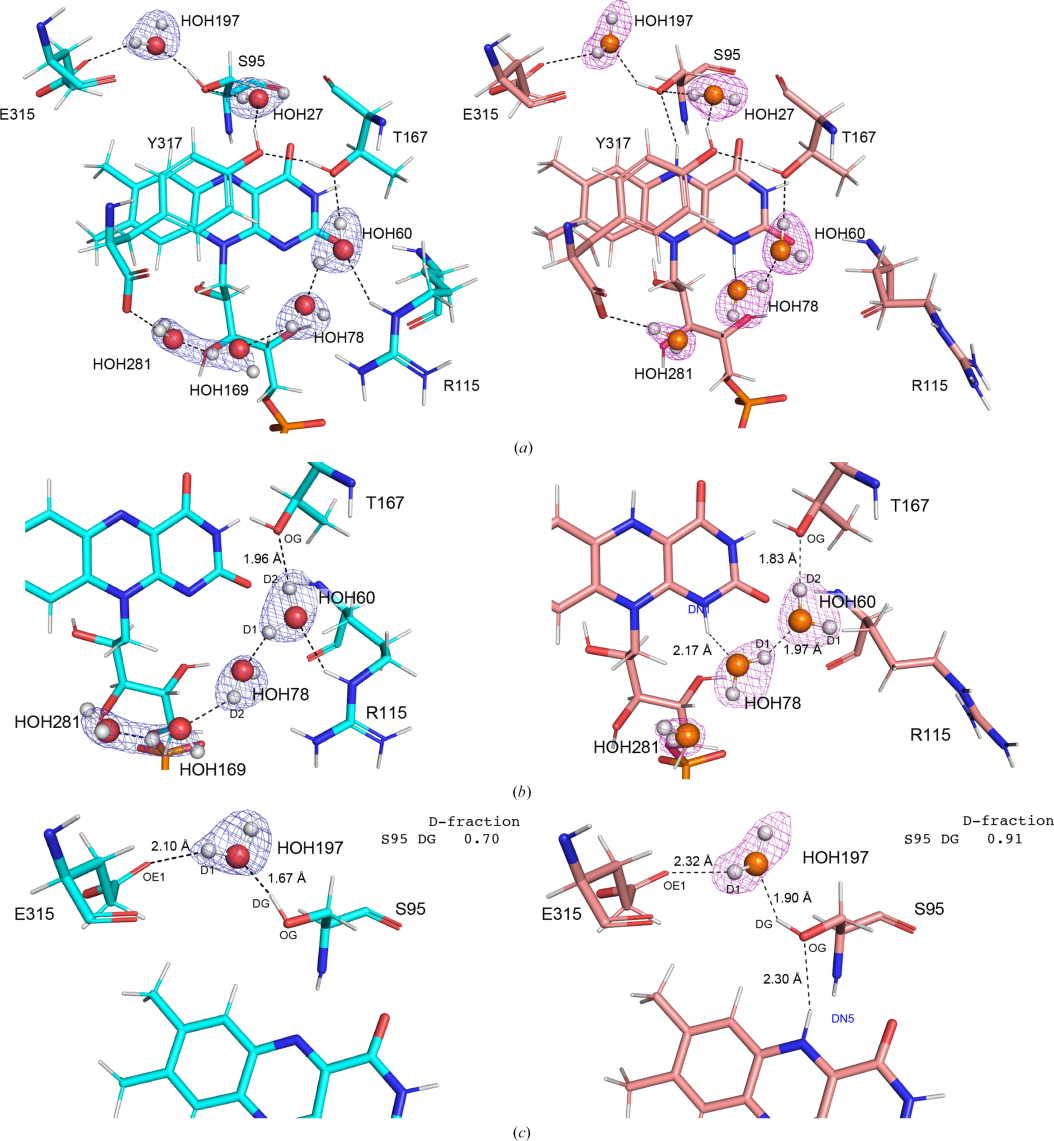
Comparison of the hydrogen-bond network around FAD/FADH_2_ between amino-acid residues and water molecules. The oxidized neutron structure (model, cyan; O atoms of water, red) is shown on the left and the reduced neutron structure (model, salmon pink; O atoms of water, orange) is shown on the right. Black dashed lines indicate hydrogen bonds. (*a*) Overview of the hydrogen-bond network. (*b*) Hydrogen-bond network focused on the DN1 atom. (*c*) Hydrogen-bond network focused on the DN5 atom.

The neutron structures of wtFNR_oxi_ and wtFNR_red_ enabled clear visualization of H atoms in water molecules, allowing a detailed description of the hydrogen-bond network in both redox states. In the wtFNR_oxi_ structure, the hydrogen-bond network around FAD involved six water molecules. These water molecules were connected through interactions with the side chains of Glu315, Ser95, Thr167, Arg115 and Tyr317 (Fig. 4 ▸*a*, left). Upon completion of the two-step reduction of FAD, accompanied by protonation at the N5 and N1 atoms of the isoalloxazine ring, two additional hydrogen bonds were formed. The first new bond, termed ‘H-bond S’, was formed between the H(D)N5 hydrogen and the O^γ^ atom of Ser95. The second bond, termed ‘H-bond R’, was formed between the H(D)N1 hydrogen and the O atom of HOH78 (Fig. 4 ▸*a*, right). These rearrangements highlight the dynamic reorganization of the hydrogen-bond network that occurs during the redox transition of FAD to FADH_2_.

## 4. Discussion

**Figure 5:**
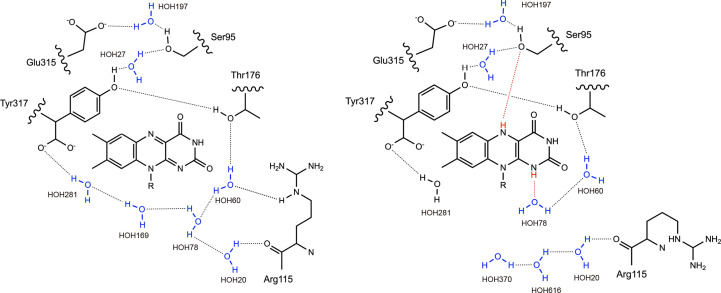
Proposed redox-dependent rearrangement of hydrogen bonds within FNR. The proposed reduction-induced rearrangement of hydrogen bonds is illustrated, highlighting important residues involved in the process.

In this study, we successfully determined high-resolution X-ray and neutron structures of R-FNR from maize in two redox states, confirming the results with micro-spectroscopy. The root-mean-square deviation (r.m.s.d.) of C^α^ atoms (residues 9–317) between wtFNR_oxi_ and wtFNR_red_ was 0.121 Å, indicating minimal structural differences at the backbone level. However, the *F*_o_(wtFNR_oxi_) − *F*_o_(wtFNR_red_) difference map revealed subtle but significant changes localized around the FAD/FADH_2_ moiety, highlighting the structural impact of FAD reduction. Key observations include increased flexibility in the Arg115 side chain upon reduction, as evident from the disappearance of side-chain electron density except for the C^β^ and C^γ^ atoms (Supplementary Fig. S6). This is consistent with a positive peak in the difference map corresponding to the missing Arg115 side chain. Additionally, reduction-induced movement of the C-terminal carboxyl group of Tyr317 caused a 0.84 Å displacement of the OXT atom, which propagated shifts in hydrogen-bonded water molecules (HOH281 and HOH169) by 1.78 and 2.03 Å, respectively. This sequential rearrangement is likely to originate from the H atom [H(D)N1] of FADH_2_ (Fig. 5 ▸). Neutron crystallography enabled the determination of hydrogen positions, providing deeper insights into the hydrogen-bond network rearrangement. In the wtFNR_red_ neutron structure, the newly positioned HOH78 disrupted its bond to HOH169 and formed a new hydrogen bond to HOH60, which subsequently lost its interaction with Arg115 (Fig. 4 ▸*b*). This cascade of events appears to be critical to increase the side-chain flexibility of Arg115 (Fig. 5 ▸) and might impact the change in Fd–FNR affinity because the electrostatic interaction of the boundary surface between Fd and FNR is a primary force in the formation of a productive electron-transfer complex. The R115A mutant (mtFNR) mimicked the dis­ordered side chain of wtFNR_red_, with its X-ray structure confirming that the water-molecule positions in mtFNR_oxi_ resemble those in wtFNR_red_. Another critical observation involved the Ser95 side chain, which rotated towards the isoalloxazine ring in wtFNR_red_. The O^γ^ atom of Ser95 was 0.32 Å closer to the isoalloxazine ring, forming hydrogen bonds through HOH27 to Tyr317 (Fig. 4 ▸*c*). This suggests increased flexibility of Tyr317 in the reduced state, potentially facilitating its displacement to accommodate the nicotinamide ring of NADP^+^. Additionally, oblique hydrogen bonds from Ser95 and HOH78 to the isoalloxazine ring could contribute to the displacement of Tyr317 by exerting stress on the FADH_2_ ring.

Despite the distinct dissociation constants of FNRs in different redox states, their backbone structures and water accessibility remained largely similar, which is supported by the comparable H/D-exchange ratios of the two neutron structures. However, neutron crystallography provided a unique advantage in visualizing redox-dependent hydrogen-bond rearrangements that are undetectable by X-ray analysis (Fig. 5 ▸). Our integrated approach revealed that small structural changes upon FAD reduction can amplify to significant disorder in the Arg115 side chain, which is likely to underpin the redox-dependent modulation of the affinity of FNR for Fd.

## Supplementary Material

PDB reference: ferredoxin–NADP^+^ reductase from maize root, oxidized form, X-ray structure, 9kkg

PDB reference: neutron structure, 9kkc

PDB reference: reduced form, X-ray structure, 9kkh

PDB reference: neutron structure, 9kk7

Preparation, crystallization and X-ray diffraction experiment for the R115A mutant and Supplementary Tables and Figures. DOI: 10.1107/S2053230X25000524/nw5131sup1.pdf

## Figures and Tables

**Table 1:** Crystallographic data and refinement statistics for X-ray structures Values in parentheses are for the highest resolution shell.

	X-ray, oxidized form	X-ray, reduced form
Data-collection statistics		
Beamline	BL44XU, SPring-8	BL44XU, SPring-8
Wavelength (Å)	0.90000	0.90000
Space group	*P*3_2_21	*P*3_2_21
Temperature (K)	100	100
*a*, *b*, *c* (Å)	59.29, 59.29, 187.22	59.08, 59.08, 186.90
α, β, γ (°)	90.00, 90.00, 120.00	90.00, 90.00, 120.00
Resolution (Å)	45.02–1.15 (1.17–1.15)	44.879–1.10 (1.12–1.10)
Total reflections	849232 (27008)	1003869 (48755)
Unique reflections	136343 (6686)	154288 (7518)
*R*_merge_	0.101 (0.351)	0.119 (0.441)
*R*_meas_	0.109 (0.404)	0.129 (0.480)
*R*_p.i.m._	0.042 (0.195)	0.049 (0.186)
CC_1/2_	0.995 (0.895)	0.992 (0.901)
Mean *I*/σ(*I*)	10.1 (3.1)	9.1 (3.6)
Completeness (%)	99.9 (99.8)	100.0 (100.0)
Multiplicity	6.2 (4.0)	6.5 (6.5)
Wilson *B* factor (Å^2^)	9.2	11.3
Refinement		
Resolution (Å)	45.02–1.15 (1.17–1.15)	44.879–1.10 (1.12–1.10)
No. of reflections	136335	154328
*R*_work_	0.1420	0.1430
*R*_free_	0.1585	0.1715
R.m.s.d. from ideal geometry		
Bond lengths (Å)	0.006	0.005
Bond angles (°)	0.948	0.952
Ramachandran plot		
Most favored (%)	99.00	99.00
Allowed (%)	1.00	1.00
Outliers (%)	0.00	0.00
PDB code	9kkg	9kkh

**Table 2:** Crystallographic data and refinement statistics for neutron structures Values in parentheses are for the highest resolution shell.

	Neutron, oxidized form	Neutron, reduced form
Data-collection statistics		
Beamline	BL03, J-PARC MLF	BL03, J-PARC MLF
Wavelength (Å)	2.05–5.38	2.05–5.38
Space group	*P*3_2_21	*P*3_2_21
*a*, *b*, *c* (Å)	59.29, 59.29, 187.22	59.32, 59.32, 187.69
α, β, γ (°)	90.00, 90.00, 120.00	90.00, 90.00, 120.00
Temperature (K)	100	100
Resolution (Å)	19.41–1.80 (1.86–1.80)	19.42–1.80 (1.86–1.80)
Total reflections	327068 (25378)	346849 (27228)
Unique reflections	36250 (3539)	36398 (3565)
*R*_merge_	0.4419 (1.8256)	0.4940 (1.9039)
*R*_p.i.m._	0.1548 (0.7180)	0.1669 (0.7279)
CC_1/2_	0.9682 (0.3492)	0.9613 (0.3668)
Mean *I*/σ(*I*)	6.34 (1.04)	5.41 (1.04)
Completeness (%)	99.5 (99.5)	99.9 (99.9)
Multiplicity	9.0226 (7.1710)	9.5293 (7.6376)
Refinement		
Resolution (Å)	19.415–1.80	19.425–1.80
No. of reflections	34484	34626
*R*_work_	0.1902	0.1971
*R*_free_	0.2438	0.2462
No. of atoms		
Total	6555	6363
Water	447	413
R.m.s.d. from ideal geometry		
Bond lengths (Å)	0.008	0.009
Bond angles (°)	1.673	1.783
PDB code	9kkc	9kk7

## References

[bb1] Adams, P. D., Afonine, P. V., Bunkóczi, G., Chen, V. B., Davis, I. W., Echols, N., Headd, J. J., Hung, L.-W., Kapral, G. J., Grosse-Kunstleve, R. W., McCoy, A. J., Moriarty, N. W., Oeffner, R., Read, R. J., Richardson, D. C., Richardson, J. S., Terwilliger, T. C. & Zwart, P. H. (2010). *Acta Cryst.* D**66**, 213–221. 10.1107/S0907444909052925 PMC2815670 20124702

[bb2] Afonine, P. V., Grosse-Kunstleve, R. W., Echols, N., Headd, J. J., Moriarty, N. W., Mustyakimov, M., Terwilliger, T. C., Urzhumtsev, A., Zwart, P. H. & Adams, P. D. (2012). *Acta Cryst.* D**68**, 352–367. 10.1107/S0907444912001308 PMC3322595 22505256

[bb3] Agirre, J., Atanasova, M., Bagdonas, H., Ballard, C. B., Baslé, A., Beilsten-Edmands, J., Borges, R. J., Brown, D. G., Burgos-Mármol, J. J., Berrisford, J. M., Bond, P. S., Caballero, I., Catapano, L., Chojnowski, G., Cook, A. G., Cowtan, K. D., Croll, T. I., Debreczeni, J. É., Devenish, N. E., Dodson, E. J., Drevon, T. R., Emsley, P., Evans, G., Evans, P. R., Fando, M., Foadi, J., Fuentes-Montero, L., Garman, E. F., Gerstel, M., Gildea, R. J., Hatti, K., Hekkelman, M. L., Heuser, P., Hoh, S. W., Hough, M. A., Jenkins, H. T., Jiménez, E., Joosten, R. P., Keegan, R. M., Keep, N., Krissinel, E. B., Kolenko, P., Kovalevskiy, O., Lamzin, V. S., Lawson, D. M., Lebedev, A. A., Leslie, A. G. W., Lohkamp, B., Long, F., Malý, M., McCoy, A. J., McNicholas, S. J., Medina, A., Millán, C., Murray, J. W., Murshudov, G. N., Nicholls, R. A., Noble, M. E. M., Oeffner, R., Pannu, N. S., Parkhurst, J. M., Pearce, N., Pereira, J., Perrakis, A., Powell, H. R., Read, R. J., Rigden, D. J., Rochira, W., Sammito, M., Sánchez Rodríguez, F., Sheldrick, G. M., Shelley, K. L., Simkovic, F., Simpkin, A. J., Skubak, P., Sobolev, E., Steiner, R. A., Stevenson, K., Tews, I., Thomas, J. M. H., Thorn, A., Valls, J. T., Uski, V., Usón, I., Vagin, A., Velankar, S., Vollmar, M., Walden, H., Waterman, D., Wilson, K. S., Winn, M. D., Winter, G., Wojdyr, M. & Yamashita, K. (2023). *Acta Cryst.* D**79**, 449–461.

[bb4] Bowsher, C. G., Dunbar, B. & Emes, M. J. (1993). *Protein Expr. Purif.* **4**, 512–518. 10.1006/prep.1993.1067 8286947

[bb6] Catapano, L., Long, F., Yamashita, K., Nicholls, R. A., Steiner, R. A. & Murshudov, G. N. (2023). *Acta Cryst.* D**79**, 1056–1070. 10.1107/S2059798323008793 PMC7615533 37921806

[bb7] Chikuma, Y., Miyata, M., Lee, Y.-H., Hase, T. & Kimata-Ariga, Y. (2021). *Biosci. Biotechnol. Biochem.* **85**, 860–865. 10.1093/bbb/zbaa102 33693505

[bb8] Deng, Z., Aliverti, A., Zanetti, G., Arakaki, A. K., Ottado, J., Orellano, E. G., Calcaterra, N. B., Ceccarelli, E. A., Carrillo, N. & Karplus, P. A. (1999). *Nat. Struct. Biol.* **6**, 847–853. 10.1038/12307 10467097

[bb9] Emsley, P., Lohkamp, B., Scott, W. G. & Cowtan, K. (2010). *Acta Cryst.* D**66**, 486–501. 10.1107/S0907444910007493 PMC2852313 20383002

[bb10] Flores, E., Frías, J. E., Rubio, L. M. & Herrero, A. (2005). *Photosynth. Res.* **83**, 117–133. 10.1007/s11120-004-5830-9 16143847

[bb11] Garman, E. F. (2010). *Acta Cryst.* D**66**, 339–351. 10.1107/S0907444910008656 PMC2852297 20382986

[bb12] Glauser, D. A., Bourquin, F., Manieri, W. & Schürmann, P. (2004). *J. Biol. Chem.* **279**, 16662–16669. 10.1074/jbc.M313851200 14769790

[bb13] Goss, T. & Hanke, G. (2014). *Curr. Protein Pept. Sci*, **15**, 385–393. 10.2174/1389203715666140327113733 PMC4030315 24678667

[bb16] Hanke, G. & Mulo, P. (2013). *Plant Cell Environ.* **36**, 1071–1084. 10.1111/pce.12046 23190083

[bb14] Hanke, G. T., Endo, T., Satoh, F. & Hase, T. (2008). *Plant Cell Environ.* **31**, 1017–1028. 10.1111/j.1365-3040.2008.01814.x 18410491

[bb15] Hanke, G. T., Kurisu, G., Kusunoki, M. & Hase, T. (2004). *Photosynth. Res.* **81**, 317–327. 10.1023/B:PRES.0000036885.01534.b8 16034535

[bb17] Hosoya, T., Nakamura, T., Katagiri, M., Birumachi, A., Ebine, M. & Soyama, K. (2009). *Nucl. Instrum. Methods Phys. Res. A*, **600**, 217–219.

[bb18] Kabsch, W. (2010). *Acta Cryst.* D**66**, 125–132. 10.1107/S0907444909047337 PMC2815665 20124692

[bb19] Kean, K. M., Carpenter, R. A., Pandini, V., Zanetti, G., Hall, A. R., Faber, R., Aliverti, A. & Karplus, P. A. (2017). *FEBS J.* **284**, 3302–3319. 10.1111/febs.14190 PMC5626627 28783258

[bb20] Kim, J. Y., Nakayama, M., Toyota, H., Kurisu, G. & Hase, T. (2016). *J. Biochem.* **160**, 101–109. 10.1093/jb/mvw016 26920048

[bb21] Kinoshita, M., Kim, J. Y., Kume, S., Lin, Y., Mok, K. H., Kataoka, Y., Ishimori, K., Markova, N., Kurisu, G., Hase, T. & Lee, Y.-H. (2017). *Biochem. Biophys. Res. Commun.* **482**, 909–915. 10.1016/j.bbrc.2016.11.132 27894842

[bb22] Knaff, D. B. (1996). *Oxygenic Photosynthesis: The Light Reactions*, edited by D. R. Ort & C. F. Yocum, pp. 333–361. Dordrecht: Kluwer Academic Publishers.

[bb23] Kurisu, G., Kusunoki, M., Katoh, E., Yamazaki, T., Teshima, K., Onda, Y., Kimata-Ariga, Y. & Hase, T. (2001). *Nat. Struct. Biol.* **8**, 117–121. 10.1038/84097 11175898

[bb24] Kurisu, G., Nishiyama, D., Kusunoki, M., Fujikawa, M., Katoh, M., Hanke, G. T., Hase, T. & Teshima, K. (2005). *J. Biol. Chem.* **280**, 2275–2281. 10.1074/jbc.M408904200 15513928

[bb25] Kusaka, K., Hosoya, T., Yamada, T., Tomoyori, K., Ohhara, T., Katagiri, M., Kurihara, K., Tanaka, I. & Niimura, N. (2013). *J. Synchrotron Rad.* **20**, 994–998. 10.1107/S0909049513021845 PMC3795571 24121355

[bb26] Kusaka, K., Yokoyama, T., Yamada, T., Yano, N., Tanaka, I. & Mizuguchi, M. (2020). *Acta Cryst.* D**76**, 1050–1056. 10.1107/S2059798320012498 33135676

[bb27] McCoy, A. J., Grosse-Kunstleve, R. W., Adams, P. D., Winn, M. D., Storoni, L. C. & Read, R. J. (2007). *J. Appl. Cryst.* **40**, 658–674. 10.1107/S0021889807021206 PMC2483472 19461840

[bb28] Niimura, N. & Podjarny, A. (2011). *Neutron Protein Crystallography: Hydrogen, Protons, and Hydration in Bio-macromolecules*. Oxford University Press.

[bb29] Ohhara, T., Kusaka, K., Hosoya, T., Kurihara, K., Tomoyori, K., Niimura, N., Tanaka, I., Suzuki, J., Nakatani, T., Otomo, T., Matsuoka, S., Tomita, K., Nishimaki, Y., Ajima, T. & Ryufuku, S. (2009). *Nucl. Instrum. Methods Phys. Res. A*, **600**, 195–197.

[bb30] Ohnishi, Y., Muraki, N., Kiyota, D., Okumura, H., Baba, S., Kawano, Y., Kumasaka, T., Tanaka, H. & Kurisu, G. (2020). *J. Biochem.* **167**, 549–555. 10.1093/jb/mvaa045 32282907

[bb31] Onda, Y., Matsumura, T., Kimata-Ariga, Y., Sakakibara, H., Sugiyama, T. & Hase, T. (2000). *Plant Physiol.* **123**, 1037–1046. 10.1104/pp.123.3.1037 PMC59067 10889253

[bb32] Sakakibara, Y., Kimura, H., Iwamura, A., Saitoh, T., Ikegami, T., Kurisu, G. & Hase, T. (2012). *J. Biochem.* **151**, 483–492. 10.1093/jb/mvs028 22427434

[bb33] Shinohara, F., Kurisu, G., Hanke, G., Bowsher, C., Hase, T. & Kimata-Ariga, Y. (2017). *Photosynth. Res.* **134**, 281–289. 10.1007/s11120-016-0331-1 28093652

[bb34] Tanaka, I., Kusaka, K., Hosoya, T., Niimura, N., Ohhara, T., Kurihara, K., Yamada, T., Ohnishi, Y., Tomoyori, K. & Yokoyama, T. (2010). *Acta Cryst.* D**66**, 1194–1197. 10.1107/S0907444910033020 21041936

[bb35] Tanaka, I., Kusaka, K., Tomoyori, K., Niimura, N., Ohhara, T., Kurihara, K., Hosoya, T. & Ozeki, T. (2009). *Nucl. Instrum. Methods Phys. Res. A*, **600**, 161–163.

[bb36] Teshima, K., Fujita, S., Hirose, S., Nishiyama, T., Kurisu, G., Kusunoki, M., Kimata-Ariga, Y. & Hase, T. (2003). *FEBS Lett.* **546**, 189–194. 10.1016/s0014-5793(03)00559-3 12832038

[bb37] Ueno, G., Kanda, H., Hirose, R., Ida, K., Kumasaka, T. & Yamamoto, M. (2006). *J. Struct. Funct. Genomics*, **7**, 15–22. 10.1007/s10969-005-9005-5 16645781

[bb38] Ueno, G., Kanda, H., Kumasaka, T. & Yamamoto, M. (2005). *J. Synchrotron Rad.* **12**, 380–384. 10.1107/S0909049505004735 15840925

[bb39] Yamashita, K., Wojdyr, M., Long, F., Nicholls, R. A. & Murshudov, G. N. (2023). *Acta Cryst.* D**79**, 368–373. 10.1107/S2059798323002413 PMC10167671 37158197

[bb40] Yano, N., Yamada, T., Hosoya, T., Ohhara, T., Tanaka, I., Niimura, N. & Kusaka, K. (2018). *Acta Cryst.* D**74**, 1041–1052. 10.1107/S2059798318012081 PMC6213574 30387763

[bb41] Yonekura-Sakakibara, K., Onda, Y., Ashikari, T., Tanaka, Y., Kusumi, T. & Hase, T. (2000). *Plant Physiol.* **122**, 887–894. 10.1104/pp.122.3.887 PMC58925 10712553

[bb5] Zeldin, O. B., Gerstel, M. & Garman, E. F. (2013). *J. Appl. Cryst.* **46**, 1225–1230.

