# Supplementary material for: Redox-dependent hydrogen-bond network rearrangement of ferredoxin–NADP+ reductase revealed by high-resolution X-ray and neutron crystallography
Source: Acta Crystallogr F Struct Biol Commun. 2025 Feb 6;81(Pt 3):73–84. doi: 10.1107/S2053230X25000524 (PMC11866413; doi:10.1107/S2053230X25000524)
Supplement: Supplementary file 1 [file f-81-00073-sup1.pdf]

STRUCTURAL BIOLOGY COMMUNICATIONS

Volume 81 (2025)

Supporting information for article:

Redox-dependent hydrogen-bond network rearrangement of ferredoxin-NADP$^+$ reductase revealed by high-resolution X-ray and neutron crystallography

Midori Uenaka, Yusuke Ohnishi, Akane Ise, Jiang Yu, Naomine Yano, Katsuhiro Kusaka, Hideaki Tanaka and Genji Kurisu

S1. Materials and Methods

S1.1. Preparation, Crystallization, and X-ray Diffraction Experiment for the R115A Mutant

The site-directed mutagenesis of R115A R-FNR was performed using polymerase chain reaction (PCR) amplification with designed primers (Table S2), employing the wild-type pQE60-R-FNR plasmid as the template, followed by DpnI digestion of the template DNA. The PCR products were transformed into *Escherichia coli* DH5α, and the R115A mutation was confirmed by plasmid sequencing. Expression and purification of the mutants were carried out using the same protocol as for wtFNR. The R115A mutant crystal was obtained using the hanging drop vapor diffusion method. The protein stock was diluted to a concentration of 30 mg/mL in a buffer containing 50 mM Tris-HCl (pH 7.5) and 150 mM NaCl and mixed with an equal volume of precipitant solution (0.2 M 2-(N-morpholino) ethane sulfonic acid, pH 6.0, and 26% PEG 2000). The mixture was equilibrated against the precipitant solution at 20 °C. After crystal growth was complete, single crystals (~200 × 300 × 100 μm^3) were soaked in a cryoprotectant solution (20% glycerol, 0.24 M 2-(N-morpholino) ethane sulfonic acid, pH 6.4, and 15% PEG 2000) for several seconds, mounted with a CryoLoops™ (Hampton Research), and cryocooled in liquid nitrogen.

X-ray diffraction experiments for the oxidized mutant were conducted at BL44XU of SPring-8. An EIGER X 16M detector was used to detect diffraction spots. A focused X-ray beam (λ=0.9000 Å) was used for the diffraction experiment. The beam size was adjusted using a 30 μm pinhole, and the beam flux was attenuated with a 1.6 mm aluminum foil. To prevent X-ray radiation damage, data were collected in six non-overlapping angular shells from a single crystal. X-ray data collection began at one end of the crystal. A total of 200 images covering 20° of rotation were collected from one assigned point. Subsequently, the measurement point was moved 60 μm towards the opposite end, and another 20 images were collected from a fresh part of the crystal. This process of crystal movement and data collection was repeated until the entire angular range was covered by six assigned exposure points. All images were processed using XDS (Kabsch, 2010), and the six datasets were scaled and merged with XSCALE. Data collection statistics were calculated using AIMLESS (McCoy *et al.*, 2007). Phasing and model refinement were performed using the same procedure as for wtFNR.

Table S1 The statistics for the X-ray crystallography of R115A mutant.

Data-collection statistics	
Beamline	SPring-8 BL44XU
Wavelength (Å)	0.90000
Space group	$P3_121$
Unit cell *a, b, c* (Å)<br>α, β, γ (°)	59.17, 59.17, 186.18,<br>90.00, 90.00, 120.00
Temperature (K)	100
Resolution (Å)	49.41-1.35 (1.37-1.35)[1]
Total reflections	481133 (13676)[1]
Unique reflections	83861 (4086)[1]
R_{merge}	0.111 (0.607)[1]
R_{meas}	0.121 (0.721)[1]
R_{pim}	0.046 (0.384)[1]
$CC_{1/2}$	0.997 (0.644)[1]
Mean $I/\sigma(I)$	8.9 (1.5)[1]
Completeness (%)	99.8 (99.9)[1]
Multiplicity	5.7 (3.3)[1]
Wilson B-factor (Å^2)	10.868
Refinement	
Resolution (Å)	49.41-1.35 (1.37-1.35)[1]
No. of reflections	
R_{work}	0.1726
R_{free}	0.1880
R.m.s.d. from ideal geometry	
Bond lengths (Å)	0.007
Bond angles (°)	0.984
favored (%)	100.00
Ramachandran Allowed (%)	0.00
Ramachandran outliers (%)	0.00

[1] Values in parentheses are for the highest resolution shells.

Table S2 Primers used for preparation of R115A FNR.

Primer name	Sequence(5'-3')
R115A-F	GTGTCGCCCGTGCAGTTTATTATGATCCTG
R115A-R	CTGCACGGGCGACACACAAGCTTCC

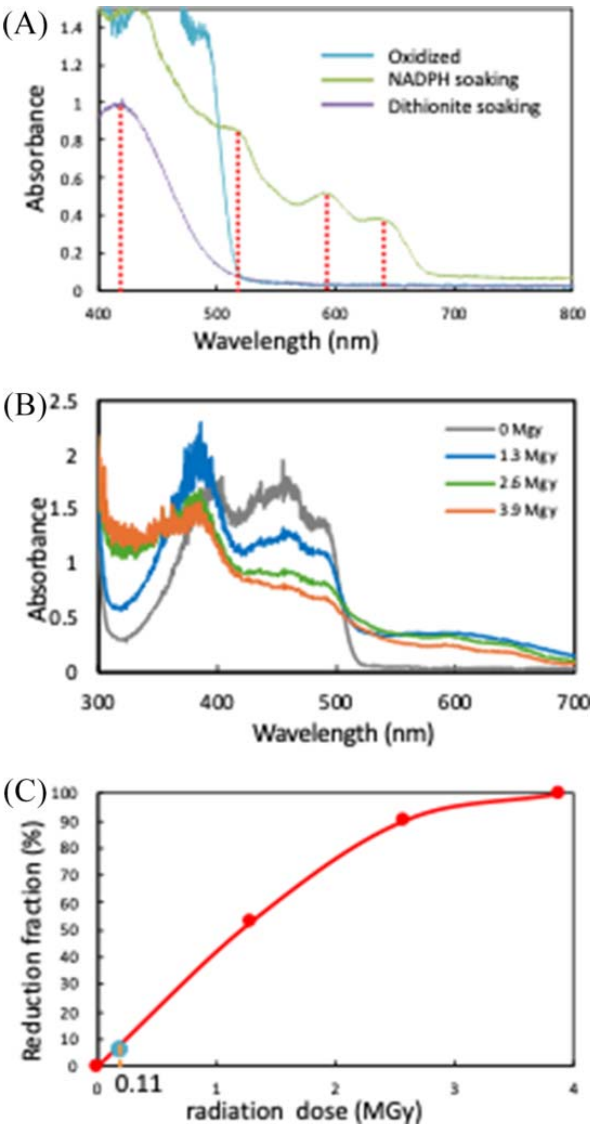

Figure S1 Absorption spectra of the R-FNR crystals by micro-spectrophotometry. (A) Absorption spectra of the R-FNR crystals with/without 5 mM dithionite and 20 mM NADPH. Ultraviolet region (<400 nm) was not shown due to the high absorbance from added dithionite. The red dashed lines indicate the characteristic peak positions in the spectra corresponding to each state. (B) Absorption spectra of the R-FNR crystal with X-ray irradiation of 0, 1.3, 2.6 and 3.9 MGy. (C) Relationship between X-ray dose and reduction fraction.

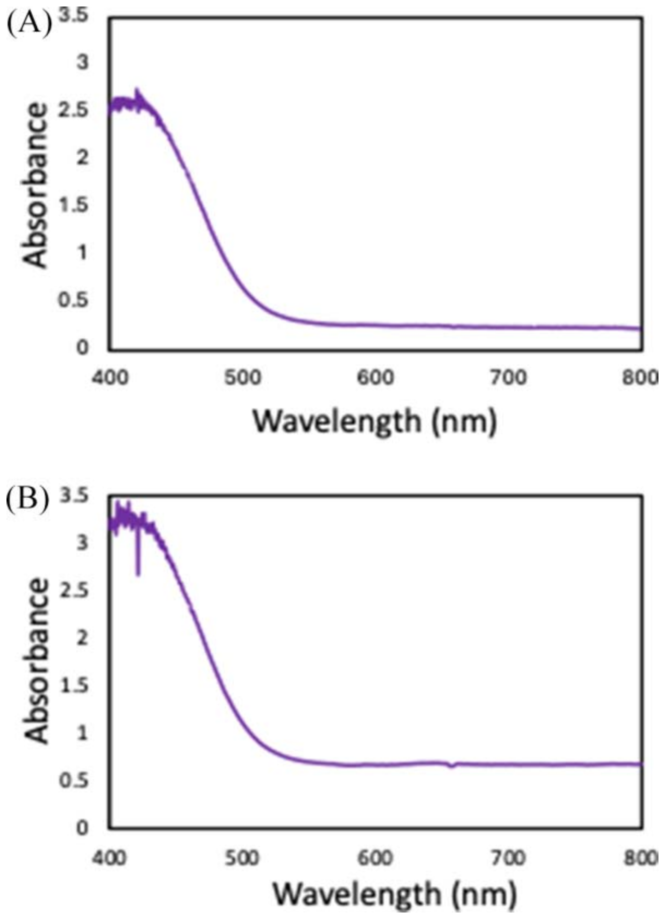

Figure S2 Absorption spectra of the large R-FNR crystals for neutron diffraction experiment by micro-spectrophotometry. Ultraviolet region (<400 nm) was not shown due to the high absorbance from added dithionite. (A) The crystal with a volume of 2.13 mm^3 (B) The crystal with a volume of 4.05 mm^3

(A)

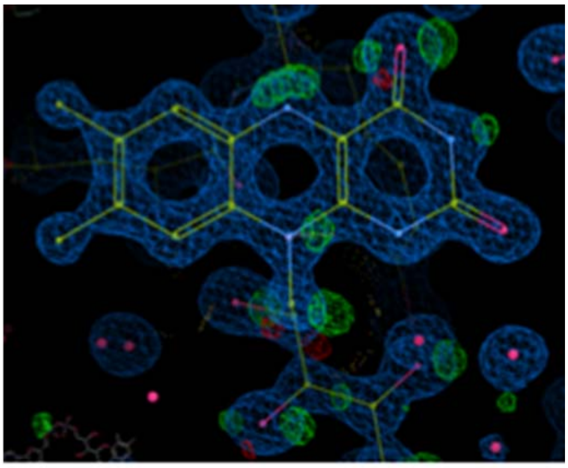

(B)

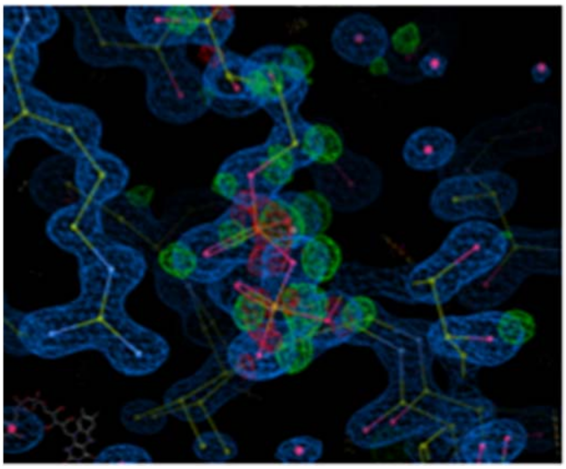

Figure S3 X-ray electron density map after refinement using anisotropic B-factors. Refined with phenix. Only FAD was selected as Anisotropic atoms in individual ADP tab. 2mFo-DFc X-ray electron density map 2.5 sigma, blue mesh. Fo-Fc omit maps of positive (green) and negative (red) peaks at counter levels of 3.52 sigma value were illustrated using Coot. (A) Around the isoalloxazine ring of FAD (B) Around the phosphoric acid of FAD

(A)

(B)

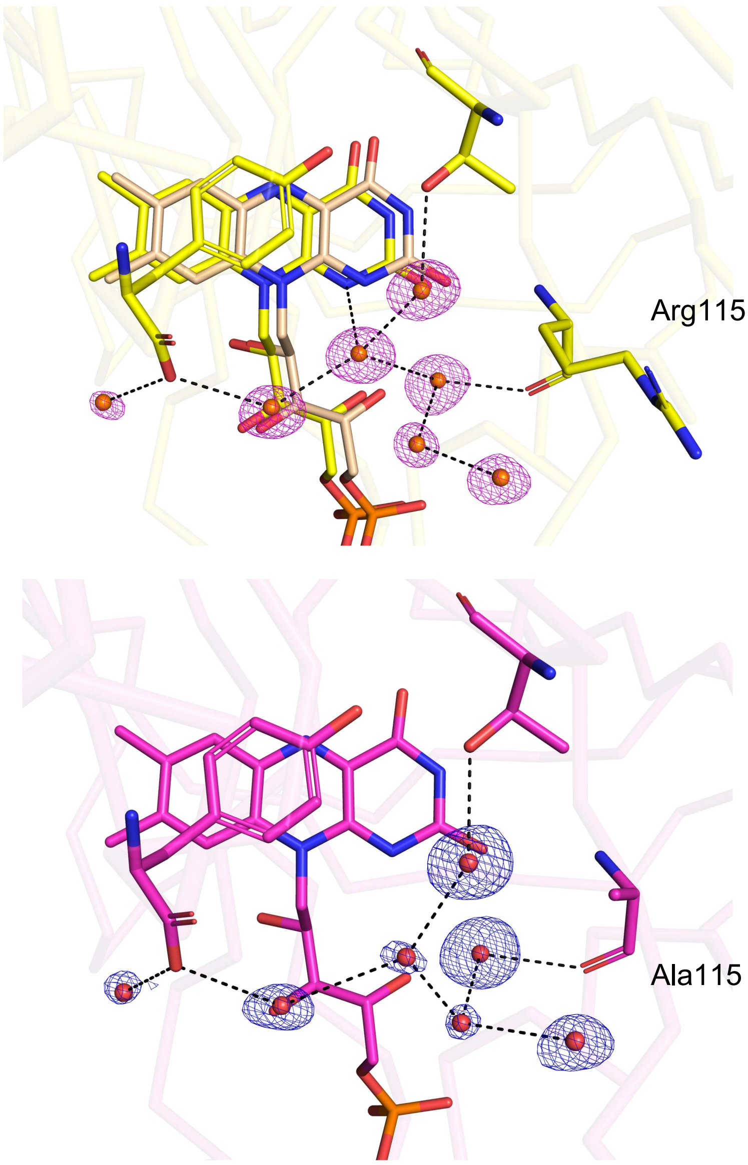

Figure S4 Water arrangement around FAD. (A) Wild-type R-FNR reduced form. Electron density (magenta mesh, 1.5σ contour level) maps for around FAD in reduced form and the structure model determined in the present study. (B) R115A mutant R-FNR oxidized form. Electron density (blue mesh, 1.3σ contour level) maps for around FAD in oxidized form and the structure model determined in the present study.

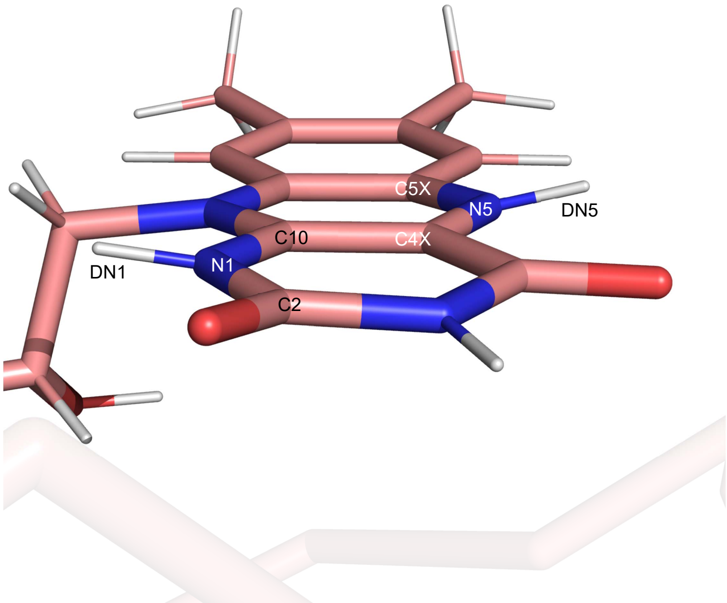

Figure S5 Angle calculation for DN1 and DN5.

Calculate two angles: θ_{DN1}, the angle between Bond DN1-N1 and Plane C2-N1-C10; θ_{DN5}, the angle between Bond DN5-N5 and Plane C4X-N5-C5X.

First, obtain two plane equations as below:

(1) Plane equation of C2-N1-C10:

$$-0.131x + 0.92y - 1.29z + 42.061 = 0$$

(2) Plane equation of C4X-N5-C5X:

$$0.339x - 0.856y + 1.336z - 43.328=0$$

Next, calculate the distance from DN1/DN5 to their corresponding plane: d_{DN1} = 0.0967Å and d_{DN5} = 0.167Å.

Then, use the bond length of DN1-N1and DN5-N5 to calculate the sine of the two angles, and finally we get θ_{DN1} = 5.53° andθ_{DN5} = 9.67°

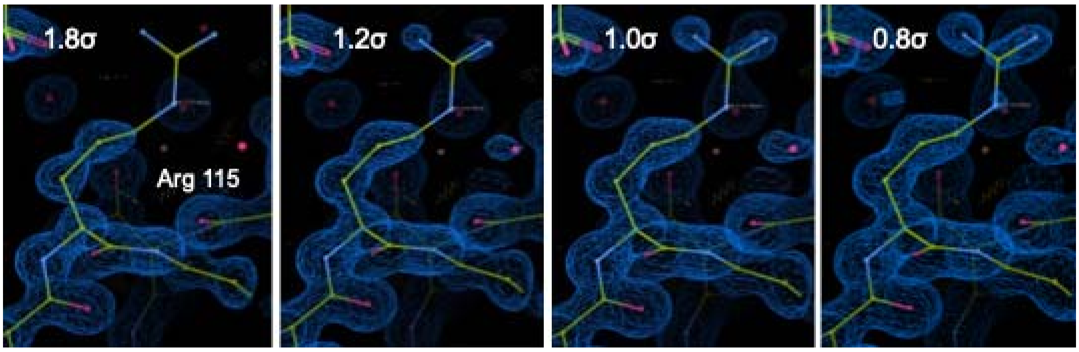

Figure S6 Electron density of R115 in the reduced X-ray structure. X-ray electron density map (blue mesh) at counter levels of indicated sigma value were illustrated using Coot.
